# Toward Inter‐Sonication Monitoring of Skull and Brain Temperatures in Transcranial MRgFUS Using a 3D Stack‐of‐Spirals Sequence

**DOI:** 10.1002/mrm.70530

**Published:** 2026-09-14

**Authors:** Sheng Chen, Steven P. Allen, John P. Mugler, G. Wilson Miller, Craig H. Meyer

**Affiliations:** ^1^ Department of Biomedical Engineering University of Virginia Charlottesville Virginia USA; ^2^ Department of Electrical and Computer Engineering Brigham Young University Provo Utah USA; ^3^ Department of Radiology and Medical Imaging University of Virginia Charlottesville Virginia USA

**Keywords:** 3D, spiral, thermometry, transcranial MRgFUS, UTE

## Abstract

**Purpose:**

To develop an MRI approach for simultaneous skull and brain temperature monitoring between sonications in transcranial MR‐guided focused ultrasound (MRgFUS) surgeries.

**Methods:**

A 3D stack‐of‐spirals dual‐echo ultra‐short TE (UTE) sequence incorporating fat suppression, spiral deblurring, trajectory measurement, and B1 correction was implemented. A variable TE strategy enabled UTEs as short as 0.05 ms for skull T1 mapping, while a fixed late‐TE (LTE) was used for brain temperature measurement. The approach was evaluated in laboratory and clinical settings through phantom and human studies.

**Results:**

During laboratory phantom cooling, MR‐estimated temperatures closely matched optical fiber measurements, with standard deviations of 0.60°C and 0.57°C across two gel ROIs. Cortical bone T1 showed a strong correlation with temperature (Pearson *r* = 0.963, *p* < 0.05; slope = 2.51 ms/°C). Similar results were obtained during laboratory phantom heating (*r* = 0.800, *p* < 0.05; slope = 3.86 ms/°C). In clinical phantom heating, gel temperature and bone T1 changed concurrently, with peak pixel‐wise temperature increases of ∼12°C and peak ROI‐averaged T1 increases of ∼14 ms. In patient scans, B1 correction reduced skull T1 spatial standard deviation from 98.1 to 59.9 ms. Volunteer uncertainty maps showed mean uncertainties of 46.8 ms for skull T1, enabling detection of ∼30 ms changes with 80% power, and 0.57°C for brain temperature.

**Conclusions:**

The proposed method enabled inter‐sonication monitoring of skull temperature changes and brain thermometry during transcranial MRgFUS. While accurate ex vivo, improvements to in vivo T1 precision are needed for clinical temperature quantification.

## 1 Introduction

Transcranial MR‐guided Focused Ultrasound (tcMRgFUS) is a minimally invasive therapy for medically refractory movement disorders. It delivers high‐intensity acoustic energy to selectively ablate targeted brain tissue by raising the temperature to 55°C–60°C [1, 2, 3]. Since the approval for the treatment of essential tremor and Parkinson's disease, clinical adoption has steadily increased worldwide [3].

In the FDA‐approved tcMRgFUS protocol, MR thermometry provides near–real‐time (∼4 s) temperature and thermal dose measurements throughout each sonication (20–30 s), enabling treatment guidance and outcome prediction [4, 5, 6]. However, monitoring is limited to a single 3‐mm‐thick imaging plane through the brain [3], acquired with body‐coil reception and multiple gradient‐echo times (3.3–27.2 ms) [7], leaving the remainder of the brain and the entire skull unobserved [8]. Unintended brain heating may arise from improper ultrasound steering [9, 10], while substantial skull heating can occur due to energy absorption during transcranial propagation, potentially causing pain and damage to the skull and adjacent brain tissue [11, 12, 13]. Ultrasound exposure may also alter skull properties, affecting treatment efficiency [10]. Although skull temperature is regulated during pauses between sonications using circulating cooled water, cooling times are typically based on simplified models that are often inaccurate, resulting in insufficient or excessive cooling and increased patient discomfort and complication risk. Therefore, a direct, noninvasive method to monitor skull heating in tcMRgFUS is critically needed. Given its whole‐skull and whole‐brain coverage, this approach is intended for use during inter‐sonication cooling periods rather than for real‐time monitoring during sonication. Since modest temperature elevations are generally clinically tolerable, precise quantification may be unnecessary. Instead, we hypothesize that T1 can serve as a proxy to detect extreme temperature changes and confirm thermal recovery, whereby a return of T1 to baseline reflects restoration of skull temperature. Under this framework, explicit calibration of the T1–temperature relationship is not required.

Measuring skull heating requires sequences distinct from those for soft tissue, as cortical bone contains little water and has an extremely short T2 [14, 15]. Ultrashort echo‐time (UTE) sequences have been explored for short‐T2 imaging [16], including cortical bone thermometry. Han et al. quantified temperature‐dependent T1 changes in cortical bone ex vivo using a 3D radial UTE sequence and the variable flip angle (VFA) method [17]. However, the 4‐min scan time with limited spatial coverage (9 × 9 × 9.6 cm^3^) may constrain clinical applicability. Ramsay et al. proposed a dual‐echo 2D GRE sequence with Cartesian trajectories to measure cortical bone signal and proton resonance frequency (PRF) shift temperatures of soft tissue [18]. However, only a single slice was scanned, and the bone signal was likely compromised by a relatively long echo time (∼1 ms) exceeding bone T2* (< 0.1 ms) [15].

Spiral and radial trajectories (e.g., stack‐of‐spirals [19] and stack‐of‐stars [20, 21]) enable very short TE and are widely used for volumetric short‐T2 imaging. Variable TE with spiral readouts further enhances UTE imaging [22]. These non‐Cartesian trajectories offer higher k‐space sampling efficiency than Cartesian methods, supporting the spatial coverage and temporal resolution demanded in clinical tcMRgFUS. Although spiral sampling is prone to blurring, particularly in interventional MRI, this can be mitigated using a retracing strategy [7, 19, 23, 24, 25], with or without a field map [24, 25, 26, 27, 28].

VFA T1 estimation relies on accurate flip‐angle calibration, which may be degraded by partial RF shielding from the conductive tcMRgFUS helmet coating [29]. Direct B1‐mapping techniques (magnitude‐ [30, 31] and phase‐based [32, 33]) assume negligible relaxation during RF excitation and before readout, which is violated in bone MRI. Alternatively, Chen et al. proposed an extrapolation‐based B1‐mapping method exploiting the spatial smoothness of the B1 field when bone is thin and adjacent to soft tissue [34]. In tcMRgFUS, the predominantly perpendicular B1 field at the skull–brain interface supports this assumption under Gauss's law, though further validation is needed.

This study employed a 3D stack‐of‐spirals dual‐echo UTE sequence integrated with fat suppression and deblurring reconstruction to enable inter‐sonication monitoring of the entire skull and brain during tcMRgFUS, addressing the aforementioned unmet need. To validate the approach, both phantom and human studies were conducted. The extrapolation‐based B1‐correction method was assessed in one patient. Assuming a linear relationship between cortical bone temperature and its T1 relaxation time, we hypothesize that, between sonications, this approach can monitor approximate skull temperature changes and their return toward baseline while concurrently measuring brain temperature.

## 2 Methods

### 2.1 Pulse Sequence Design

The pulse sequence, implemented on both Siemens and GE platforms, employed a stack‐of‐spirals trajectory to enable accelerated 3D acquisition. To capture signals from both short‐T2 and conventional‐T2 tissues, each TR included two gradient echoes: a UTE and a conventional or late TE (LTE), as illustrated in Figure 1. For phantom studies on the Siemens platform, both echoes utilized a spiral‐out readout. For volunteer scans on Siemens and for clinical implementation using RTHawk (Vista.ai, Palo Alto, CA) with a GE scanner, the first echo employed a spiral‐out readout, while the second echo used a spiral retraced‐in‐out (RIO) readout. The RIO trajectory spirals inward to the k‐space center and then immediately reverses along the same path, causing off‐resonance phase to accrue in opposite directions, facilitating robust image deblurring and accurate PRF phase estimation [24]. In all implementations, the spirals were designed with constant density to minimize trajectory imperfection. Parameters are provided in Table 1.

**FIGURE 1:**
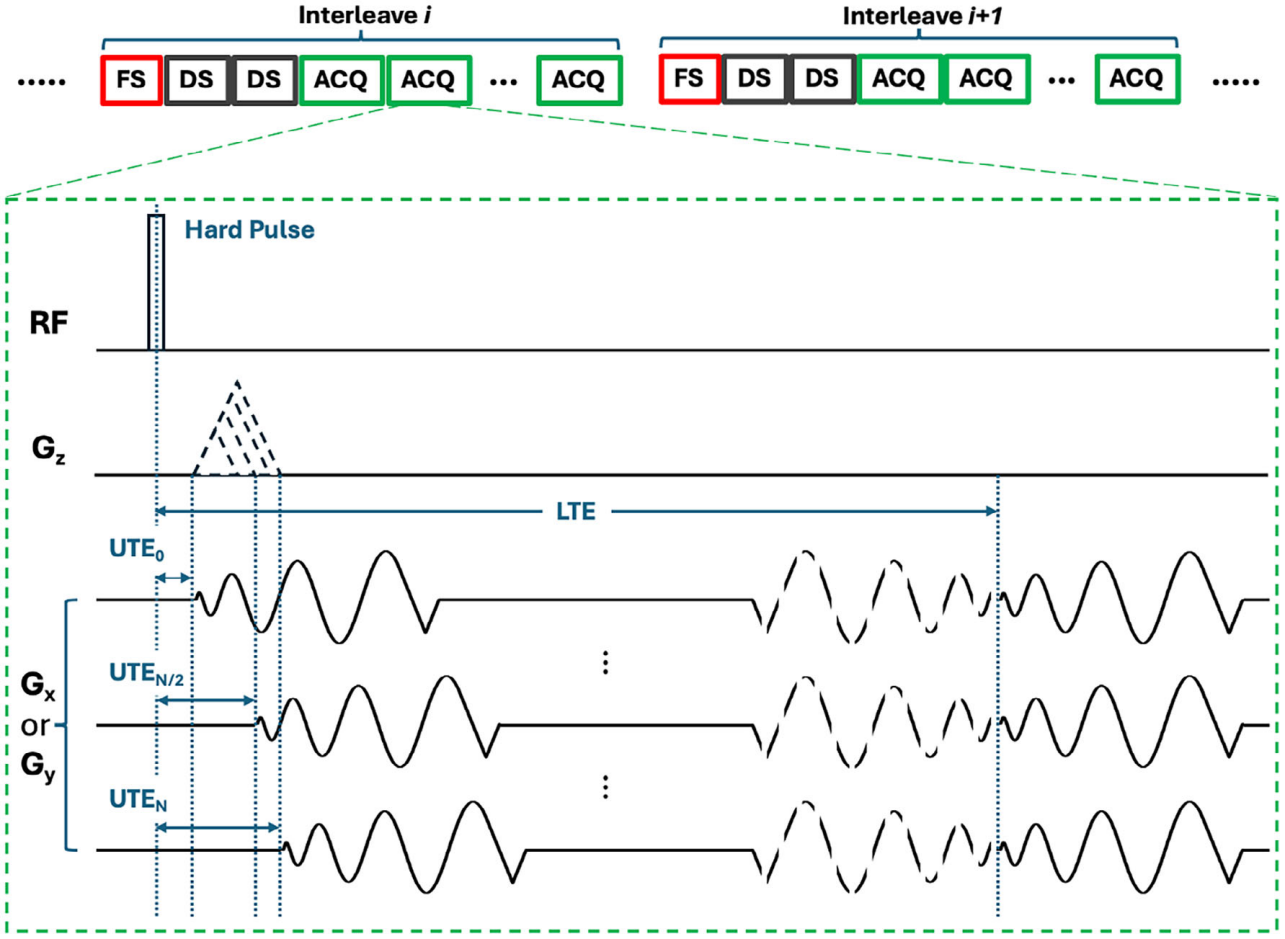
The pulse sequence diagram illustrates the 3D dual‐echo stack‐of‐spirals sequence implemented. An effective UTE as low as 0.05 ms was achieved using a short nonselective hard RF pulse, variable duration kz encoding gradient, and a spiral‐out readout. On the clinical platform and for human scans, the spiral RIO gradients center at LTE, whereas for the laboratory phantom studies, the second echo was acquired using a spiral‐out readout only, omitting the initial spiral‐in segment. For clarity, the Gz rewinder, in‐plane and through‐plane spoiler gradients, and RF spoiling are not shown. ACQ, acquisition; DS, dummy scan; FS, fat suppression.

**TABLE 1:** Acquisition parameters for the phantom and human scans used in this study.

Parameter	TR (ms)		First echo		Second echo						
		Spiral inter‐leaves	TE (ms)	In‐plane trajectory	TE (ms)	In‐plane trajectory	Resolution (mm)	FOV (cm)	Nominal flip angle (°)	Frame period for both FAs (s)	Fat suppressionpulse
Phantom study (lab)	30	64	0.05 (cooling), 0.07 (heating)	Spiral‐out	10	Spiral‐out	1.35 × 1.35 × 8	13 × 13 × 8	15, 30	72	N/A
Phantom study (clinical)	54.6	24	0.18	Spiral‐out	17	Spiral RIO	2.5 × 2.5 × 5 (first echo), 2 × 2 × 5 (second echo)	34 × 34 × 29.5	10, 36	56 (w/sliding window)	N/A
Patient and volunteer	24.6	25	0.18	Spiral‐out	17	Spiral RIO	2 × 2 × 5	30 × 30 × 29.5	10, 36	89	SPAIR

To achieve a UTE as low as 0.05 ms, a short‐duration hard RF pulse was used. The kz encoding gradients were designed with variable durations: zero for the zeroth encoding step and non‐zero for other steps [22, 35]. The spiral readouts of the first echo were initiated immediately following the kz encoding gradients and began at the center of k‐space, thereby minimizing the effective TE. The variable TEs act as a low‐pass filter along the partition encoding direction of k‐space. While this is generally not problematic for the first echo, as only the magnitude of the rapidly decaying bone signal is required, the second echo may suffer from phase deviations that further degrade PRF accuracy, particularly in the presence of steep variations in precession frequency induced by temperature changes [36, 37]. To mitigate this, a constant TE was used for the second echo across all kz encodings.

Flip angles were chosen to balance the requirements of the VFA method and the UTE scheme. Prior studies [38, 39] suggest that, for a single tissue, optimal precision in VFA‐based T1 estimation is achieved using two flip angles that yield approximately 71% of the signal obtained at the Ernst angle [40]. Assuming TR = 25 ms and T1 = 240 ms for cortical bone [41], the theoretically optimal flip angles were approximately 11° and 56°. While the smaller angle was readily implemented, the larger angle would require an RF pulse duration, given RF‐amplifier and specific absorption rate (SAR) constraints, that is too long for effective bone imaging, resulting in excessive residual signal from nonselective excitation of both the head and the water in the tcMRgFUS transducer that would be difficult to fully suppress. Consequently, to accommodate the 3D UTE acquisition constraints, the larger flip angle was reduced to 35°.

### 2.2 Fat Suppression

Fat suppression near the skull is essential for detecting cortical bone signal, as fat has a higher proton density than bone [42]. Spectral adiabatic inversion recovery (SPAIR), a hybrid method that incorporates both relaxation and spectral selectivity, was selected over chemical shift‐selective fat suppression (CHESS) and spectral presaturation with inversion recovery (SPIR) [43] due to reduced sensitivity to B_1_ inhomogeneity in tcMRgFUS. It was also preferred over short inversion time inversion recovery (STIR) for its higher SNR, as STIR relies solely on relaxation behavior [43] and may not adequately distinguish fat from cortical bone given their comparable T1 values [44].

Given scan time and SAR constraints, SPAIR was applied periodically before each zeroth kz encoding as an optional module in the sequence, which was structured with kz steps as the inner loop and spiral interleaves as the outer (Figure 1). To establish a steady state at the zeroth kz step, SPAIR timing was calculated from the selected flip angles using the Bloch equations, with appropriate dummy scans included.

### 2.3 Spiral Deblurring

Blurring in spiral MRI generally arises from off‐resonance effects and trajectory infidelity caused by susceptibility, hardware imperfection, eddy‐current effects, and concomitant fields [26, 27, 28, 45]. At field strengths of 1.5–3 T used in tcMRgFUS, field inhomogeneity is often the dominant source, as treatment hardware introduces additional field perturbations. Fortunately, knowledge of the field heterogeneity can be exploited for deblurring by estimating a field map from the first two frames, using a 1.8‐ms offset in the second‐echo TE (similar to Marx et al. [46]). The map was used for off‐resonance correction during non‐Cartesian gridding reconstruction [47], employing a semiautomatic method [26] for spiral‐out data and an energy‐based method [7, 25] for RIO data.

To further reduce blurring, trajectory corrections were applied on both GE and Siemens systems. On GE, in‐plane trajectories were modeled using zeroth‐, first‐, and second‐order gradient impulse response function (GIRF) [48] terms for each gradient axis. On Siemens, k‐space trajectories were estimated by the Tan‐Meyer method [45]. During offline 3D MATLAB (MathWorks, Natick, MA) reconstruction, measured trajectories were incorporated into 2D non‐Cartesian gridding after inverse Fourier transform along the partition‐encoding direction.

### 2.4 T1 Mapping

VFA‐based T1 mapping [49] was performed using linear regression with two flip angles based on

$$\frac{S}{\sin\alpha }=E_{1}\frac{S}{\tan\alpha }+M_{0}\left(1-E_{1}\right)E_{2}$$

where S is signal intensity, α is the flip angle, $M_{0}$ is equilibrium magnetization, $E_{1}=e^{-\frac{\mathrm{TR}}{T_{1}\left(T\right)}}$, and $E_{2}=e^{-\frac{\mathrm{TE}}{T_{2}\left(T\right)}}$. Treating $M_{0}E_{2}$ as a single unknown allows T1 to be extracted from two flip‐angle measurements. The method was applied pixelwise to UTE‐reconstructed images. Bone masks were generated using scaled subtraction [22] and manual refinement.

### 2.5 PRF Temperature Mapping

PRF‐based temperature mapping [36, 37] was applied pixelwise to LTE‐reconstructed images using

$$\Delta T=\frac{\Delta \theta }{\alpha \gamma B_{0}T_{E}}$$

where ΔT is temperature change, Δθ is phase change, and α is the thermometry coefficient (−0.00909 ppm/°C). The first frame was discarded, and the second frame served as the baseline. Spatial phase unwrapping was applied as needed, and mean temperatures in unheated regions were subtracted from each frame to correct for global drift during heating experiments and human scans.

### 2.6 Variable TE Simulation

Numerical simulations were performed in MATLAB to evaluate the impact of fluctuating partition‐encoding durations on PRF thermometry accuracy. A 3D digital object (128 × 128 × 128 mm^3^, T2* = 40 ms) with a 30°C maximum 3D Gaussian temperature hotspot was simulated at 3 T, using a minimum LTE of 10 ms and 64 kz encodings.

The independent variables tested were spatial resolution (1 and 4 mm) and gradient hardware performance. To simulate realistic hardware constraints, two gradient systems were compared: a high‐performance system (80 mT/m maximum amplitude, 200 T/m/s slew rate) and a low‐performance system (20 mT/m, 40 T/m/s).

The methodology involved calculating gradient‐limited variations in partition‐encoding durations to determine partition‐specific TEs for each combination of spatial resolution and gradient hardware. Temperature‐induced phase shifts were applied using the standard PRF model, and T2*‐related signal decay was incorporated to account for signal loss at longer TEs. Temperature maps were reconstructed from the simulated data and compared to a ground‐truth reference. Artifacts and accuracy loss were quantified by measuring the peak temperature and full‐width‐at‐half‐maximum (FWHM) of the reconstructed hotspot.

### 2.7 Laboratory Phantom Experiments

To demonstrate feasibility, two phantom experiments were conducted using a 3 T scanner (MAGNETOM Prisma‐Fit, Siemens Healthineers, Erlangen, Germany) in a laboratory setup. In each experiment, the phantom consisted of agar gel surrounding an ex vivo bovine femur with marrow removed. An oversampling rate of 60% was used to reduce the signal from water outside the field of view (FOV). Detailed sequence parameters are listed in Table 1.

In the cooling experiment (Figure 2A), three optical fibers were positioned in cortical bone, inner gel, and outer gel. The phantom was fully immersed in hot water (∼65°C, prepared by cooling freshly boiled water) until thermal equilibrium, which was monitored with an optical thermometer (L201, Rugged Monitoring, Quebec City, Canada). A commercial transmit/receive head coil was used, and the sequence was acquired in six measurements of four repetitions each, with 20‐min intervals. The RF pulse duration was 60 μs, resulting in a UTE of 0.05 ms.

**FIGURE 2:**
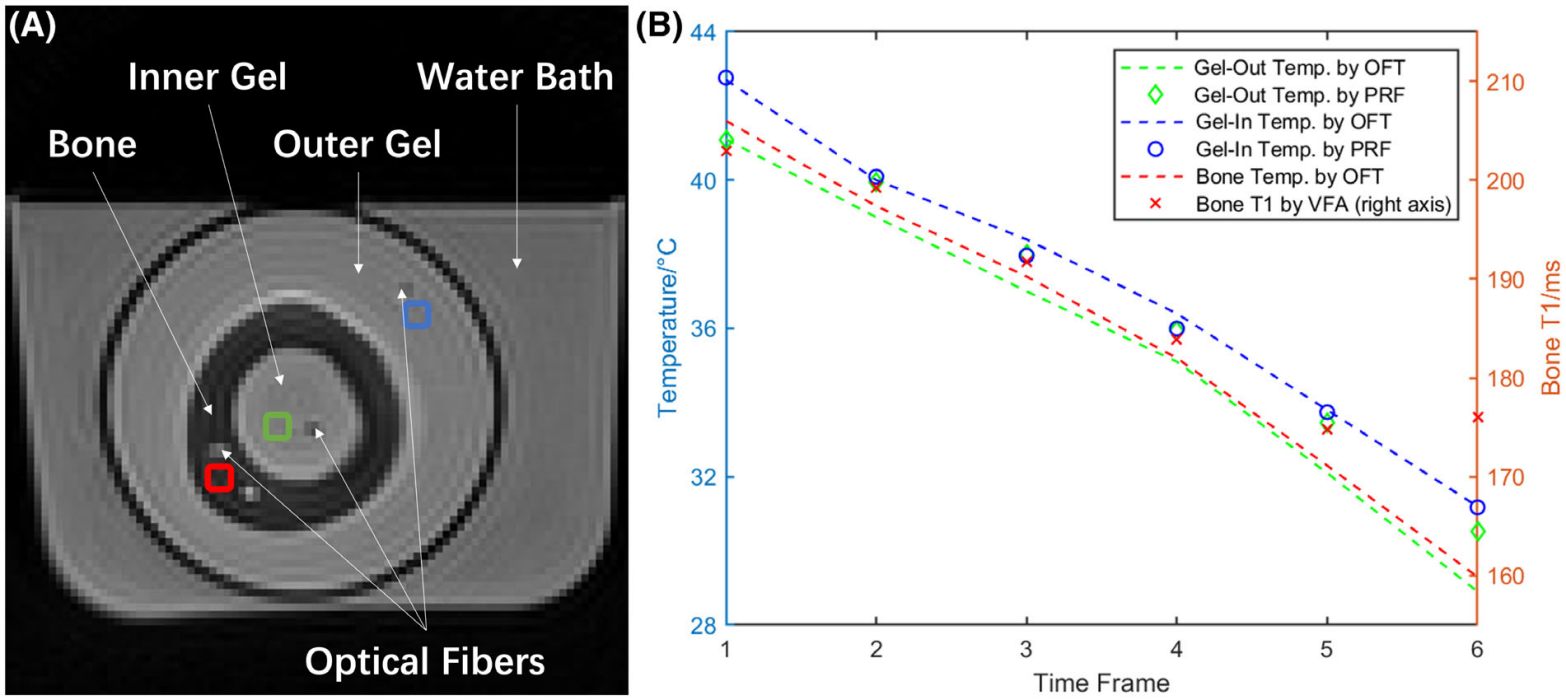
Laboratory cooling experiment. (A) Experimental setup. A phantom consisting of cortical bone embedded in agar gel on both the inner and outer sides was used. 4 × 4 pixel ROIs were selected in the bone (red), inner gel (green), and outer gel (blue), each near an optical fiber. (B) Results demonstrate accurate PRF thermometry and a linear relationship between temperature and T1. Data were smoothed over four scans and averaged across the ROIs shown in panel (A). OFT denotes an optical fiber thermometer.

In the heating experiment (Figure 3A), the phantom was placed in a bottomless plastic cylinder to allow ultrasound to reach the bone–gel interface vertically. An optical fiber was inserted in the gel ∼2 cm from the acoustic focus, which was targeted with 5 W focused ultrasound sonications at a frequency of 1.13 MHz for 114 s (LP‐100, FUS Instruments, Toronto, Canada). A commercial ring‐shaped flexible surface coil was positioned around the cylinder for signal reception. The RF pulse duration was 100 μs, resulting in a UTE of 0.07 ms.

**FIGURE 3:**
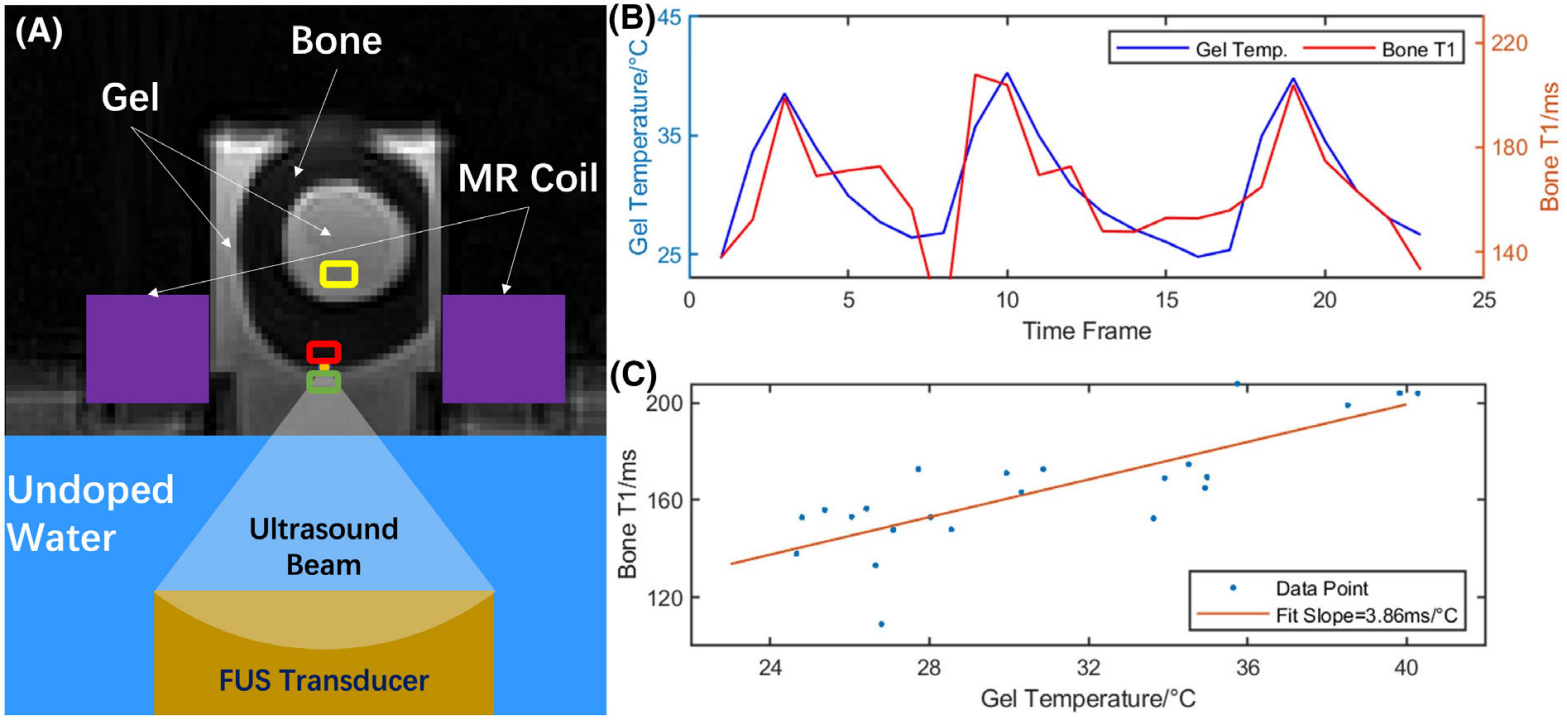
Laboratory FUS‐heating experiment. (A) Experimental setup. A phantom consisting of cortical bone embedded in agar gel on both the inner and outer sides was used. Two 3 × 5 pixel ROIs (red and green) were selected from bone and gel regions near the yellow‐marked acoustic focus at the bone–gel boundary, and a 4 × 6 pixel ROI (yellow) was selected as an unheated region for PRF drift correction. (B) Results demonstrate that T1 closely tracked temperature over three consecutive heating and cooling cycles. Data were averaged over the ROIs shown in panel A. (C) A scatter plot demonstrates a strong positive correlation between PRF temperatures and VFA T1 values.

Post‐reconstruction analysis was performed on regions of interest (ROIs) of 4 × 4 pixels for the cooling experiment and 3 × 5 pixels for the heating experiment using the VFA and PRF methods alongside thermometer readings.

### 2.8 Phantom Heating in Clinical Setup

Another phantom FUS‐heating experiment was performed using a clinical tcMRgFUS system (ExAblate Neuro, InSightec, Haifa, Israel) and a 3 T scanner (MR750, General Electric, Waukesha, WI). A small bone–gel phantom was molded in a hemispheric container repurposed from a daily quality assurance (DQA) phantom (Figure 4A). Because the phantom was positioned within the tightly sealed transducer for ultrasound delivery through water, no optical fiber was included. The water between the phantom and transducer was degassed to < 2 ppm dissolved oxygen and cooled to 15°C. Focused ultrasound (5.5 W, 60 s) was applied immediately after the second frame of image acquisition, targeting the bone–gel boundary. The body coil was used for both transmission and reception, consistent with clinical tcMRgFUS protocols.

**FIGURE 4:**
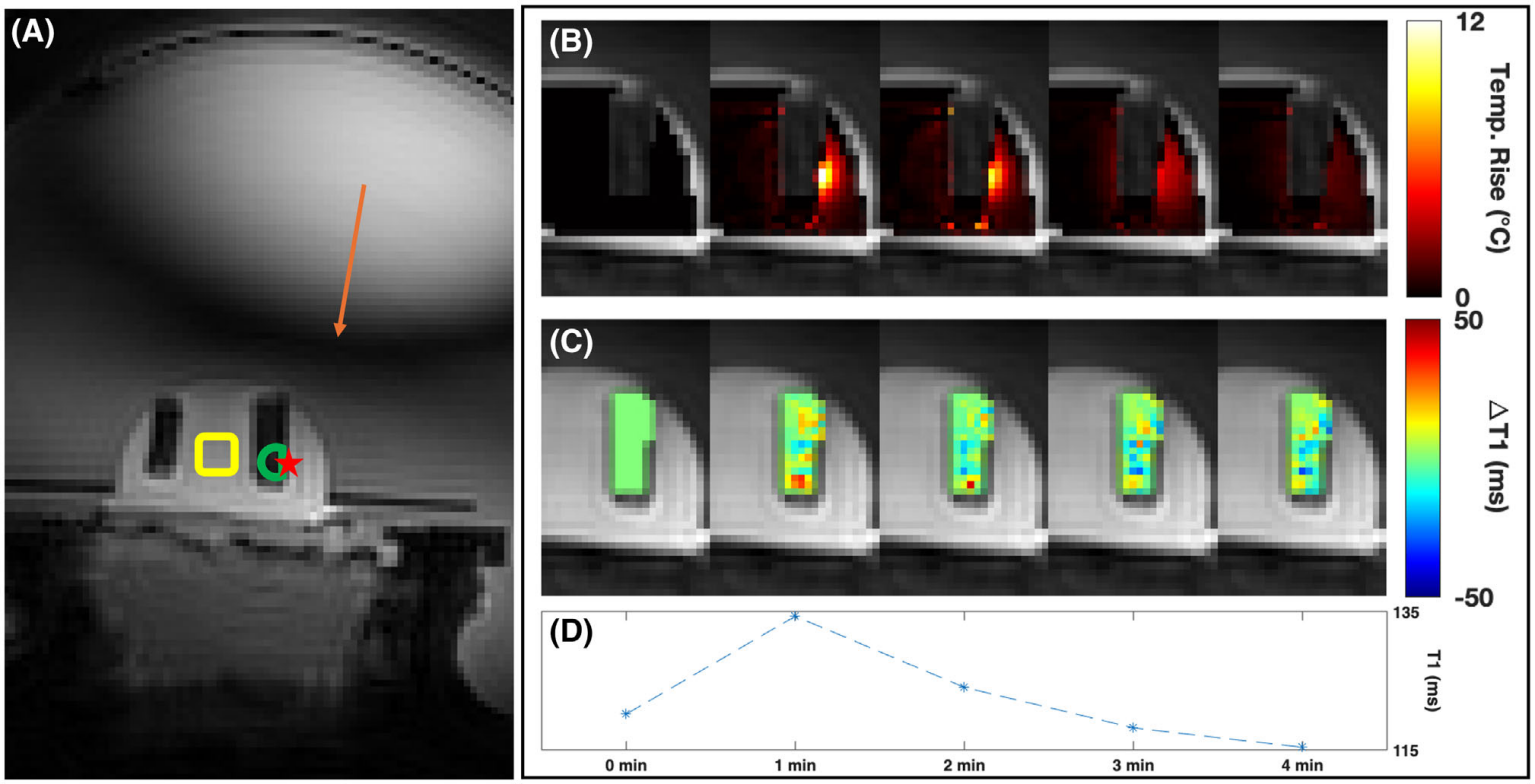
Clinical FUS‐heating experiment setup and results. (A) Experimental setup. A small hemispherical phantom containing cortical bone (dark regions) embedded in agar gel was placed within the hemispherical FUS transducer. The arrow indicates B1 inhomogeneity within the enclosed space. A yellow‐highlighted region was selected as an unheated reference for PRF drift correction, and the red star indicates the location of the focal spot. A semicircular ROI (green) in the cortical bone was used to determine the baseline T1 and quantify temporal T1 changes. (B) Zoomed‐in temperature maps of the agar gel near the focal spot during heating and cooling. (C) Corresponding ΔT1 maps in the adjacent cortical bone during heating and cooling, calculated using a pixel‐wise baseline derived from the first frame. (D) Temporal change of the spatially averaged T1 within the ROI.

The sequence, developed with RTHawk, employed a nonselective RF pulse of 160 μs duration, immediately followed by a spiral‐out readout to achieve a UTE of 0.18 ms. The second echo utilized a spiral RIO readout with a fixed LTE of 17 ms (18.8 ms for the first frame). A prolonged TR of 54.6 ms, combined with RF and gradient spoiling, allowed additional water‐signal relaxation. Twenty‐four constant‐density spiral interleaves were divided into three groups for sliding‐window acceleration, resulting in a temporal resolution of 1 min. Additional sequence parameters are provided in Table 1.

### 2.9 Patient Scans

Post‐treatment scans of two patients were included in this study with the approval of the Institutional Review Board (IRB) and informed consent. Scanning began after the patients finished all of their treatment scans of focused ultrasound thalamotomy (∼5 min after final sonication), as described by Elias et al. [1] Subject 1 was scanned in the coronal plane and Subject 2 in the sagittal plane. After the scans, both patients resumed standard post‐treatment care with their physician.

The sequence parameters are listed in Table 1. SPAIR was applied to suppress the fat signal near the skull. The scan time for a single cycle, comprising two flip angles, was 89 s. Owing to the heavy clinical constraints, each scan consisted of three cycles.

Prior to scanning Subject 1 with the proposed acquisition, a commercial 2D B1‐mapping sequence was performed in the coronal orientation. Imaging parameters were TR/TE = 30 ms/12.8 ms, TA = 40.3 s, FA = 20°, FOV = 30 cm, in‐plane resolution = 4.7 × 4.7 mm^2^, and 21 slices of 5‐mm thickness covering 20.5 cm. The resulting B1 maps were interpolated to match the spatial resolution of the proposed sequence and subsequently used to rescale the prescribed flip angles on a pixel‐wise basis for the VFA analysis.

### 2.10 Volunteer Scans

To obtain uncertainty maps for both T1 and PRF‐based temperature measurements, a prolonged 20‐cycle scan of a healthy volunteer was performed on a 3 T scanner (MAGNETOM Prisma‐Fit, Siemens Healthineers, Erlangen, Germany) under IRB approval and informed consent. To replicate the patient scans as closely as possible, the body coil and identical sequence parameters (Table 1) were used, and the same reconstruction and analysis methods were applied. Unlike the patient scans, no tcMRgFUS equipment was involved.

## 3 Results

### 3.1 Variable TE Simulation

TEs and temperature‐induced phase shifts along the kz encoding direction are shown in Figure 5A,B, respectively. Because the readout is initiated immediately after the partition‐encoding gradient to minimize dead time, higher kz steps require longer gradient durations, causing the actual TE to increase. In each simulated scenario, TEs and signal phases progressively deviate from the zeroth kz step, which corresponds to the center of k‐space and determines the primary phase contrast of the 30°C peak temperature, as the spatial resolution increases or the gradient performance degrades. Maximum deviations occur at the highest kz encoding step, ranging from 0.24 ms in TE, 0.058 rad in phase or 2.4% (at 4‐mm resolution with high gradient performance) to 1.1 ms in TE, 0.26 rad in phase or 11% (at 1‐mm resolution with low gradient performance).

**FIGURE 5:**
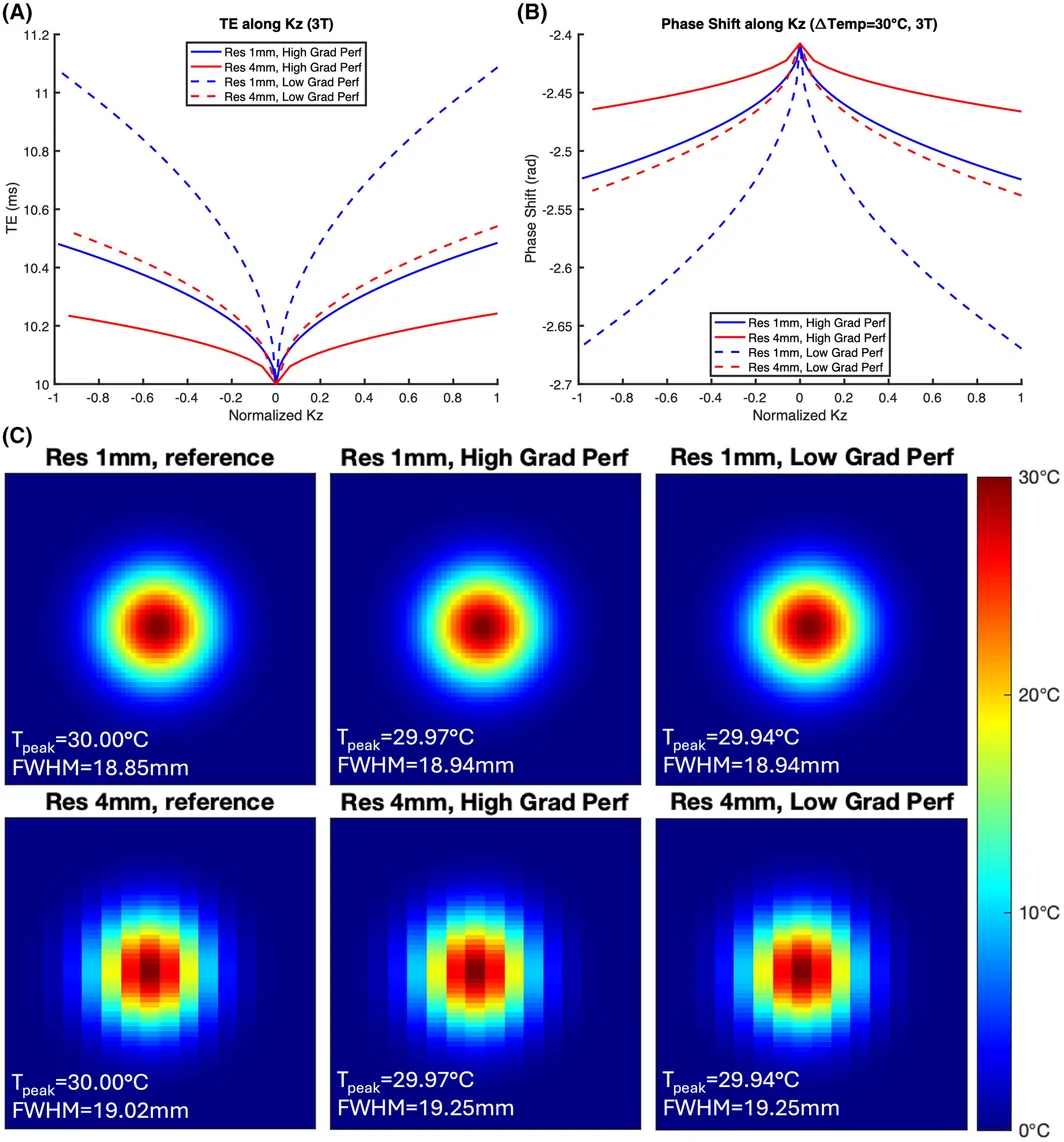
Simulation results with variable TE for PRF thermometry. TEs (A) and temperature‐induced phase shifts (B) deviate progressively from the zeroth kz encoding step. The Gaussian‐shaped temperature distributions illustrate reduced peak temperature and broadened FWHM across the four simulated scenarios (C).

The object simulation results, presented in Figure 5C, demonstrate the effects of these scenarios on peak temperature and FWHM. Overall, the simulated temperature fields exhibit blurring, characterized by reduced peak temperatures and broadened FWHM. Relative to their respective baselines, the peak temperature decreases by 0.03°C for the high‐gradient system and by 0.06°C for the low‐gradient system, while the FWHM increases by 0.09 mm at 1‐mm resolution and by 0.23 mm at 4‐mm resolution.

While these effects are minor relative to the precision of PRF thermometry, the use of a fixed second TE eliminated them.

### 3.2 Laboratory Phantom Experiments

In the cooling experiment, temperatures measured by the optical fibers, serving as the ground truth, exhibited a cooling trend from approximately 42°C to 30°C, together with a spatial temperature gradient decreasing from the inner gel to the bone and subsequently to the outer gel (Figure 2B). The T1 values calculated in bone followed a similar temporal trend. The time interval between scan frames was approximately 20 min.

Both PRF temperatures and VFA T1 values were averaged over the corresponding 4 × 4 ROIs (Figure 2A) and smoothed across four measurements. All six PRF temperature measurements in the inner and outer gel deviated by no more than 1.5°C from the ground truth, with standard deviations of 0.60°C and 0.57°C, respectively. A strong positive correlation was observed between the optical fiber temperatures and VFA T1 values in bone (Pearson *r* = 0.963, *p* < 0.05), with a fitted slope of 2.51 ms/°C.

In the heating experiment, because the acoustic focus was positioned at the bone–gel boundary, PRF temperatures in the gel were used as the reference for the VFA T1 measurements. Both datasets were averaged over 3 × 5 ROIs selected in the gel and adjacent bone near the focal zone (Figure 3A). The PRF data were averaged over every two frames to temporally align with the VFA data. The resulting curves show good agreement (Figure 3B). Consistently, Figure 3C demonstrates a strong positive correlation between PRF temperatures and VFA T1 values, with a linear fit yielding a slope of 3.86 ms/°C and a Pearson correlation coefficient of 0.800 (*p* < 0.05).

### 3.3 Phantom Heating in Clinical Setup

The entire transducer was included within the scan FOV. A dark band–shaped region is observed (arrow in Figure 4A), extending into the interior of the hemispherical transducer and resembling the artifact reported by Yan et al. [29]. This observation suggests the necessity of B1 mapping in tcMRgFUS when using the VFA method.

Figure 4B shows magnified maps illustrating temporal changes in gel temperature and bone T1 at a 1‐min temporal resolution. The PRF temperature maps clearly depict heating followed by cooling along the gel boundary, with a peak temperature increase of ∼12°C in the second frame.

The adjacent cortical bone exhibits ΔT1 maps with temporal variations that closely track the temperature changes (Figure 4C). The temporal evolution of the absolute T1 values is illustrated in Figure 4D, which plots the spatially averaged T1 within the bone ROI (Figure 4A) over time. This ROI analysis shows a peak T1 increase of ∼14 ms from the baseline, defined as the pixel‐wise T1 in the first frame.

### 3.4 Patient Scans

The original VFA T1 maps of the skull, averaged over the last two cycles and acquired without B1 correction, from three consecutive slices (each separated by a 2.5‐cm gap) for Subjects 1 and 2 are shown in Figure 6. The regions near the metal frame used in the tcMRgFUS treatment are marked with yellow boxes, within which the skull T1 maps showed significant deviations from values reported in the literature. These deviations are most likely caused by frame‐induced susceptibility artifacts. The regions of low RF excitation, indicated by dark bands, are marked with red boxes. Primarily due to the low SNR in these areas, the VFA method produced T1 values with pronounced deviations. For Subject 1, the mean ± standard deviation of skull T1 values (excluding the yellow and red regions) were 149 ± 74.0 ms, 169 ± 69.3 ms, and 272 ± 173 ms across the three slices; for Subject 2, the corresponding values were 142 ± 108 ms, 242 ± 95.8 ms, and 211 ± 68.8 ms. An overall standard deviation of 98.1 ms was observed. Partial volume effects may be the primary cause of these deviations, due to the thin cortical layer of the skull.

**FIGURE 6:**
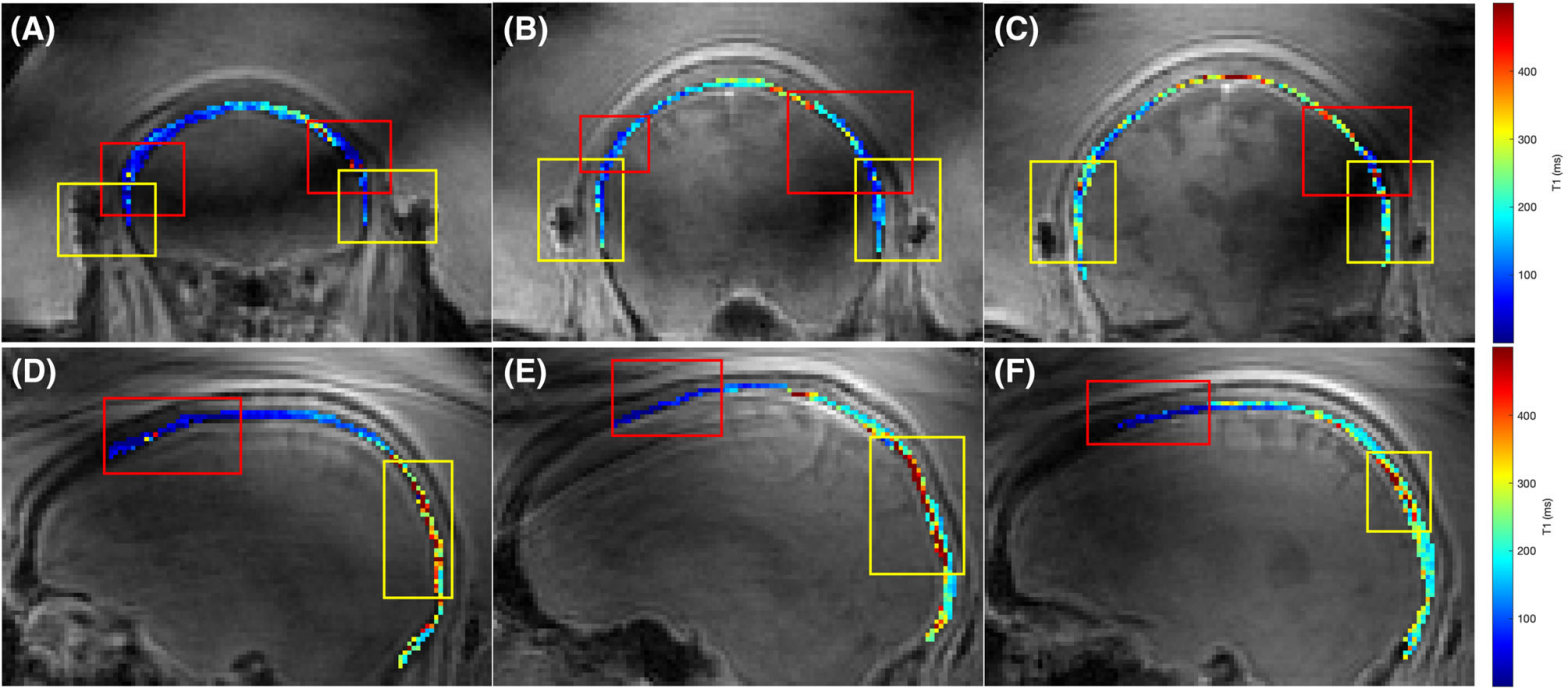
VFA T1 maps of the skull from three consecutive slices, each separated by a 2.5‐cm gap, for Subject 1 (A–C) and Subject 2 (D–F). No B1 correction was applied. All images were cropped for display. Yellow boxes highlight regions with significant T1 deviations primarily due to frame‐induced susceptibility artifacts, while red boxes indicate areas with pronounced deviations mainly attributed to B1‐related low SNR.

Correction of VFA T1 using the extrapolation‐based B1‐mapping method [34] was evaluated on two slices of Subject 1 (shown in Figure 6B,C), with results presented in Figure 7. The T1 maps outside the yellow and red boxes, which denote the same types of artifact regions as in Figure 6, were smoothed and corrected toward literature values [41] using B1 correction. The spatial variability of T1 was reduced to an overall value of 59.9 ms, with the mean ± standard deviation for each slice improving from 169 ± 69.3 ms and 272 ± 173 ms to 137 ± 53.2 ms and 137 ± 66.5 ms, respectively. However, no significant improvement was observed within the boxed areas.

**FIGURE 7:**
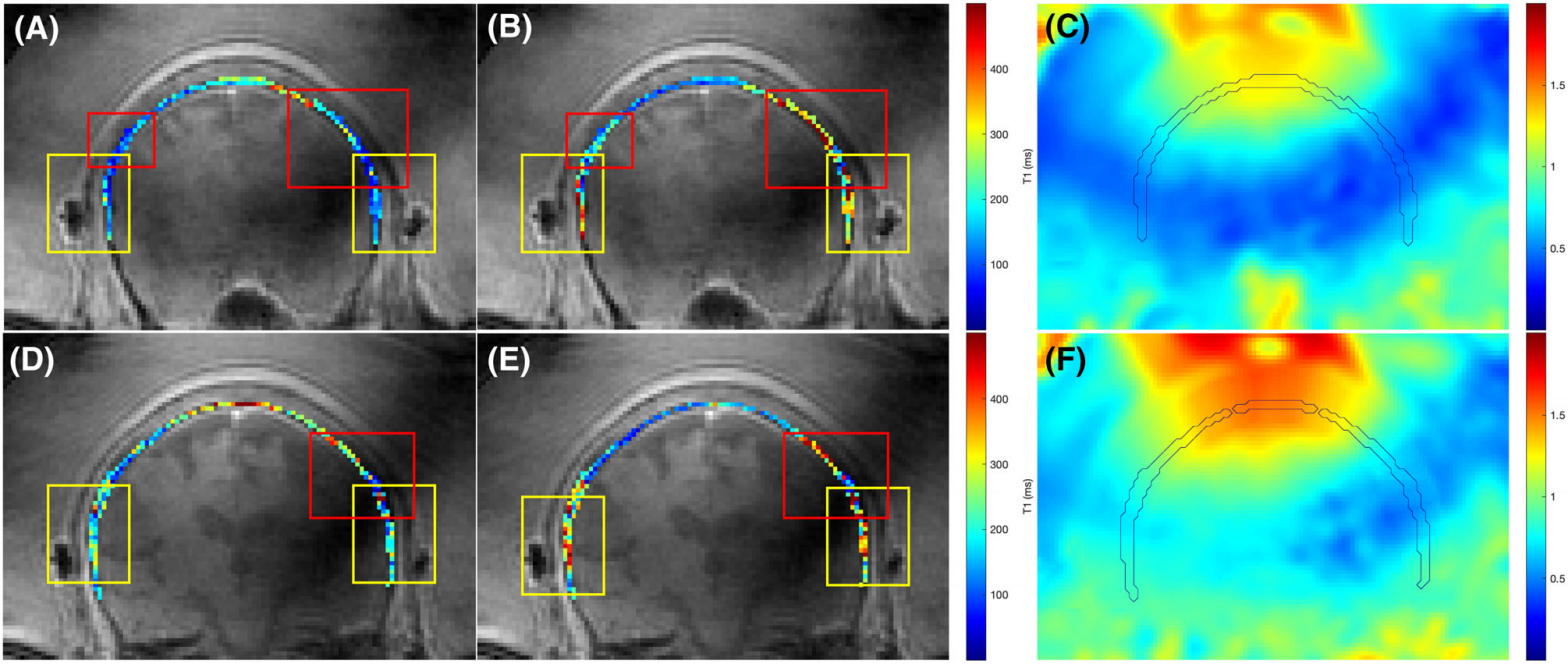
VFA T1 maps of the skull from two slices of Subject 1, shown without (A, D) and with (B, E) B1 correction, alongside the corresponding B1 maps with the skull outlined (C, F). Each row (A–C, D–F) corresponds to one slice. All images were cropped for display. The yellow and red boxes denote the same types of artifact regions as in Figure 6.

PRF temperature‐difference maps for a single frame, along with corresponding example magnitude images from two slices of each subject, are shown in Figure 8. Temperature differences were calculated from the two‐FA acquisition of the final cycle only. Within the green circles that were selected near the tcMRgFUS treatment targets, the spatial standard deviations were 0.88°C, 0.90°C, 0.77°C, and 0.50°C for Figure 8A–D, respectively. These values, with a mean of 0.76°C, are comparable to those reported by Allen et al. from Cartesian and spiral RIO scans [7].

**FIGURE 8:**
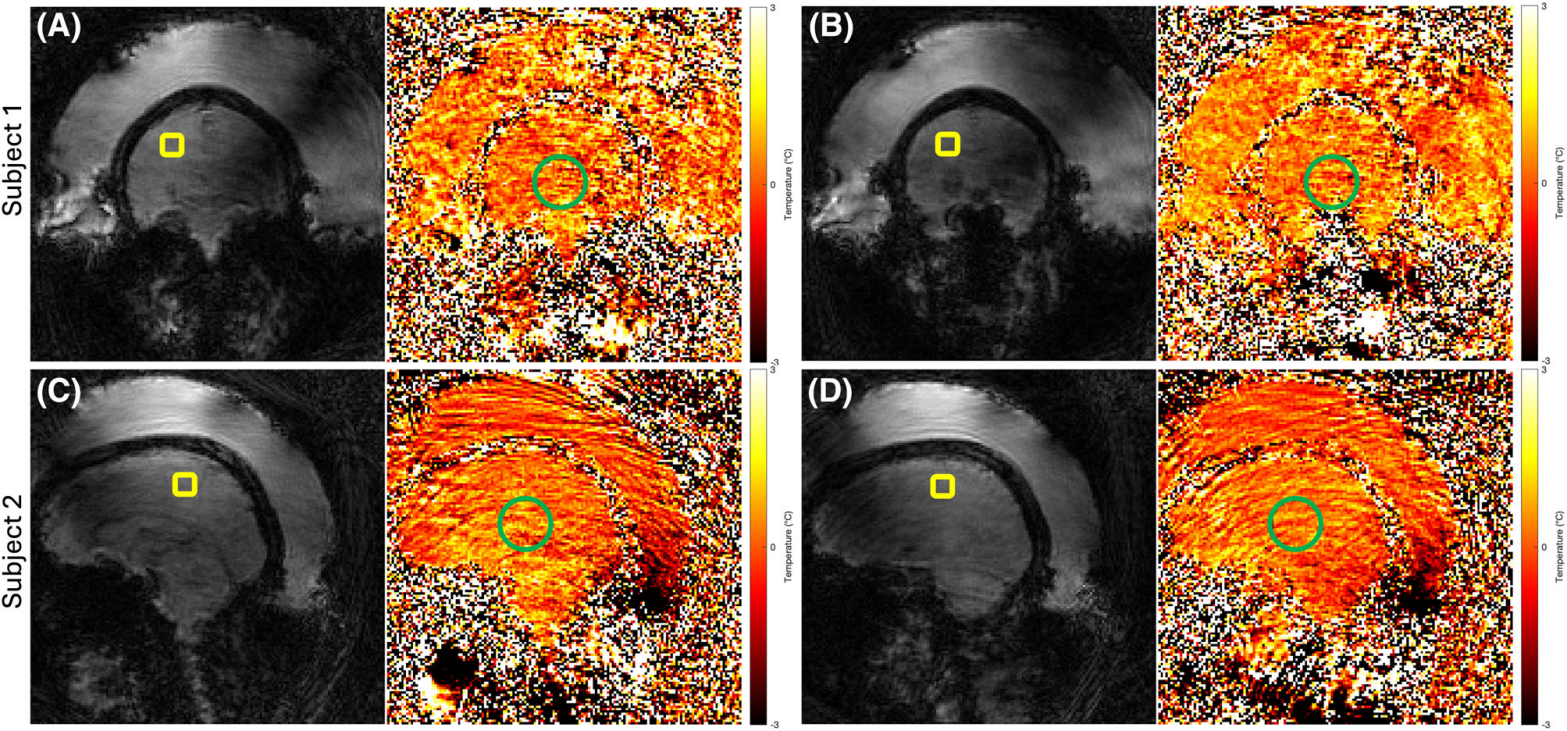
Temperature‐difference maps for a single frame and corresponding example magnitude images from two slices of Subject 1 (A, B) and Subject 2 (C, D). Green circles denote regions of interest used for statistical analysis, while the yellow boxes indicate areas used for drift correction.

### 3.5 Volunteer Scans

Uncertainty maps for VFA‐based skull T1 measurements and PRF‐based brain temperature measurements were generated from volunteer scans. For each pixel, uncertainty was quantified as the standard deviation across 20 independent repeated measurements. Representative maps are shown in Figure 9, with mean ± standard deviation of 46.8 ± 81.1 ms for whole‐skull T1 and 0.57°C ± 0.25°C for brain temperature within the green ROI.

**FIGURE 9:**
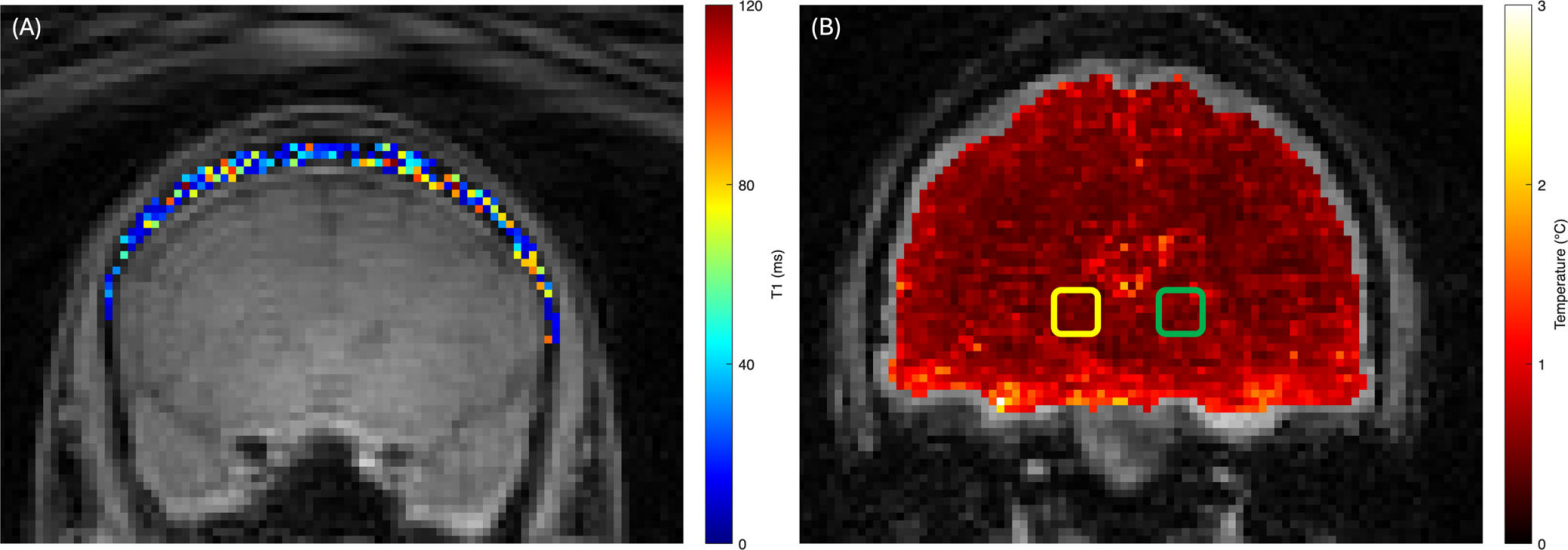
Uncertainty maps of VFA‐based T1 and PRF‐based temperature measurements from volunteer scans. Mean uncertainties were 46.8 ms for whole‐skull T1 and 0.57°C for brain temperature within the green ROI. The yellow box indicates the region used for field drift correction.

These pixel‐wise T1 uncertainty estimates were used to approximate the measurement variability relevant for detecting mean T1 differences at the group level. Under this variability, the study had 80% power to detect a mean T1 difference of ∼30 ms (two‐sided *t*‐test, α = 0.05).

## 4 Discussion

We present a novel approach for inter‐sonication monitoring of the whole skull and brain using a 3D dual‐echo UTE sequence with stack‐of‐spirals sampling and fat suppression. The reconstruction incorporates measured trajectories and applies deblurring, enabling VFA‐based skull T1 mapping and PRF‐based brain temperature mapping. Simulations reveal substantial phase deviations in high‐order kz data, underscoring the necessity of a fixed TE for accurate PRF thermometry. To systematically validate this approach for potential clinical translation, we adopted a stepwise study design. First, laboratory phantom experiments using local coils and independent temperature measurements confirmed the underlying physics of the method through cooling and heating processes. During cooling, estimated temperatures closely aligned with the ground truth, with standard deviations of 0.60°C and 0.57°C across the two gel ROIs. A strong positive correlation was observed between temperature and cortical bone T1, with a Pearson correlation coefficient of 0.963 (*p* < 0.05) and a linear fit slope of 2.51 ms/°C. Similar results were obtained during heating, demonstrating a strong correlation (Pearson *r* = 0.800, *p* < 0.05) and a comparable linear fit slope of 3.86 ms/°C. Next, to verify that these temperature‐dependent changes could be detected under realistic hardware constraints without specialized local coils, a phantom heating experiment was conducted using the integrated body coil within a clinical tcMRgFUS system. Here, gel temperature and bone T1 at the boundaries increased and decreased concurrently, with a peak PRF‐measured temperature increase of ∼12°C and a peak ROI‐averaged VFA‐derived T1 increase of ∼14 ms. Finally, patient scans performed in the full clinical tcMRgFUS setup and volunteer scans on a standard scanner using the body coil demonstrated successful in vivo implementation of the proposed approach. Without and with B1 correction, patient skull T1 maps exhibited spatial standard deviations of 98.1 and 59.9 ms, respectively. With B1 correction, T1 values within skull regions free of susceptibility artifacts and low B1 more closely matched literature values [41]. Patient brain temperatures were measured with a spatial standard deviation of 0.76°C. Volunteer uncertainty maps showed mean uncertainties of 46.8 ms for skull T1 and 0.57°C for brain temperature, enabling detection of ∼30 ms T1 change with 80% statistical power.

Based on the slopes observed in our phantom calibration experiments (2.51 and 3.86 ms/°C), the mean volunteer T1 uncertainty of 46.8 ms corresponds to an apparent temperature uncertainty of approximately 12°C–19°C. Similarly, a 30 ms T1 detection threshold corresponds to an 8°C–12°C change. However, because the T1–temperature conversion coefficient may vary biologically [50], a shallower patient‐specific slope (e.g., ∼1.5 ms/°C) would increase this detection threshold to ∼20°C. Consequently, the current precision is insufficient for accurate, degree‐level temperature quantification in patients. Nevertheless, during clinical tcMRgFUS, the skull can retain substantially more heat than the adjacent brain due to its high ultrasonic absorption, low thermal conductivity, and limited vascular perfusion, potentially reaching localized temperatures exceeding ablative thresholds (> 60°C), while the nearby brain remains comparatively cooler. Given the magnitude of these worst‐case temperature elevations in bone, a method with an approximate detection threshold of ∼20°C may still provide practical value by indicating whether skull temperature remains elevated or has returned toward the physiological baseline (∼37°C), thereby offering a potential safety margin against thermal injury.

To our knowledge, this is the first study to examine simultaneous whole‐skull and whole‐brain thermometry between FUS sonications. Achieving this required addressing constraints inherent to the clinical tcMRgFUS environment, including spatial coverage, temporal resolution, and the presence of the water‐filled transducer assembly. The sequence developed for our clinical setup was designed to encompass the entire transducer, patient skull, and brain, with each scan frame lasting 89 s to enable volumetric T1 and temperature mapping. Unlike prior studies, which did not require fat‐saturation pulses in laboratory experiments, our sequence incorporates periodic SPAIR pulses for each spiral interleave. With appropriately ordered kz encoding, these pulses ensure effective steady‐state fat suppression (Figure 1). Han et al. [17] used flip angles of 8° and 44° optimized for accurate VFA T1 quantification, although residual signals from long‐T2 tissues may complicate such measurements. In the present context, precise T1 estimation is not essential; rather, skull T1 derived from our approach reflects temperature changes and indicates when temperatures return to baseline following sonications. Ramsay et al. [18] employed a 2D dual‐echo sequence for T1‐weighted imaging of cortical bone and PRF temperature mapping; however, their longer UTEs (∼1 ms) may have reduced bone specificity due to contamination from long‐T2 signals. In contrast, our sequence uses a non‐selective hard RF pulse, stack‐of‐spirals trajectories, and variable kz encoding durations, achieving UTEs of 0.05–0.18 ms, comparable to the 0.175 ms reported by Han et al. [17], thereby enabling robust, bone‐specific T1 mapping.

Limitations exist in this study. First, unspoiled residual signal from long‐T2 tissues (primarily water) persists, particularly in the 36°‐FA images, which may degrade the accuracy of skull T1 measurements. Since this flip angle is already lower than the theoretically optimized value (> 50°), further reduction may not be feasible without compromising the balance between VFA T1 precision and image quality. A potential strategy is to selectively excite the region of interest using double‐half‐RF pulses for UTE imaging [51]; however, this approach incurs an additional time penalty that must be carefully considered in sequence optimization. Alternatively, spatial saturation pulses may be employed to suppress the water signal, although their performance may be constrained by low transmit efficiency and significant B1 inhomogeneities within the transducer and water bath [52]. Other options include T2‐preparation or inversion pulses; however, these would prolong acquisition, disrupt bone signal steady‐state, and risk exceeding SAR limits with the existing SPAIR pulses. Second, orientation constraints are present when scanning with the proposed sequence. Using a non‐selective RF pulse may cause aliasing artifacts from signals outside the FOV, such as the patient's shoulders, if the partition encoding direction aligns with the superior–inferior axis. The other two orientations were examined in each of the patient scans. Thirdly, the experiments conducted in the clinical setup rely on the accuracy of PRF‐based temperature measurements derived from laboratory results and lack temperature measurements from another source (e.g., optical fibers) as ground truth. Unfortunately, obtaining such reference measurements may be challenging due to the sealed transducer. Fourth, although the B1 maps reduced T1 deviations overall, they were ineffective in correcting VFA T1 values within regions affected by artifacts and RF‐corruption‐induced low SNR, as the B1‐correction method is inherently image‐based. Finally, patient scans were limited to three frames due to clinical workflow constraints. Although uncertainty maps were derived from 20‐cycle volunteer scans on a separate scanner with the sequence closely matched to the patient protocol, several factors could still influence direct comparison and uncertainty characterization, including slight differences in field strength, body coil gain and SNR, shimming, and potential RF banding or coupling with the transducer and water bath. Additional patient scan repetitions, in combination with in vivo heating experiments and accelerated imaging, would further strengthen the validation of the proposed approach.

## 5 Conclusion

A 3D dual‐echo UTE sequence with spiral‐out and RIO trajectories, fat‐suppression pulses, and B1 correction was implemented for simultaneous skull and brain temperature monitoring during inter‐sonication periods in tcMRgFUS treatments. Laboratory and preliminary clinical experiments demonstrated the feasibility of tracking approximate temperature trends and their return toward baseline. PRF‐based thermometry was robustly validated against reference measurements in ex vivo studies, and a strong linear relationship between VFA‐derived T1 and temperature was observed under these conditions. While patient and volunteer studies suggest potential for clinical translation, the current precision of T1 measurements may be insufficient for accurate temperature quantification in vivo. Future work will focus on mitigating long‐T2 signal contamination to improve T1 accuracy and incorporating additional patient data and in vivo heating measurements.

## Funding

This work was supported by the National Institutes of Health (R01EB028773), the Focused Ultrasound Foundation, and Siemens Healthineers (Projects UVA‐2021‐MR‐29‐01‐Mugler_C00239406 and UVA‐2025‐MR‐34‐01‐Mugler_CLMA229599.).

## Conflicts of Interest

Craig Meyer and John Mugler declare research grant funding from Siemens Healthineers.

## Supporting information


**Figure S1:** Original (A) and deblurred (B) coronal UTE images from a selected slice of Subject 1, illustrating the deblurring effect achieved by the semiautomatic method. Original (C) and deblurred (D) sagittal RIO images from a selected slice of Subject 2, demonstrating the enhanced clarity near the skull produced by the energy‐based method. Fat signal near the skull was largely suppressed in all images.

## Data Availability

Research data are not shared.
