# Supplementary material for: Toward Inter‐Sonication Monitoring of Skull and Brain Temperatures in Transcranial MRgFUS Using a 3D Stack‐of‐Spirals Sequence
Source: Magn Reson Med. 2026 Sep 14;97(1):17–30. doi: 10.1002/mrm.70530 (PMC13746072; doi:10.1002/mrm.70530)
Supplement: Supplementary file 1 [file MRM-97-17-s001.docx]

**Supporting Information**

A pronounced deblurring effect was observed in the reconstruction from patient scans obtained using the described method. Figure S1A and S1B compare the original and deblurred coronal UTE images of Subject 1, showing enhanced clarity in the skull and subcutaneous tissue. Figure S1C and S1D present sagittal RIO images of Subject 2 before and after deblurring, highlighting additional improvement near the skull achieved through the energy-based method^25^ in conjunction with the RIO acquisition strategy. Across Figure S1, fat signal between the scalp and skull was largely suppressed. Signal magnitude variations were attributed to RF inhomogeneity, and susceptibility-related artifacts were observed near the interfaces with the silicone membrane and the head frame. Residual spiral-related artifacts primarily originated from the water supply tubes and the very small portion of the 30cm-diameter coupling bath outside the FOV.

**Figure S1. Original (A) and deblurred (B) coronal UTE images from a selected slice of Subject 1, illustrating the deblurring effect achieved by the semiautomatic method. Original (C) and deblurred (D) sagittal RIO images from a selected slice of Subject 2, demonstrating the enhanced clarity near the skull produced by the energy-based method. Fat signal near the skull was largely suppressed in all images.**
